# Correlation Between Nitrous Oxide and Functional Vitamin B12 Deficiency Resulting in Subacute Combined Degeneration of the Spinal Cord: A Case Report

**DOI:** 10.7759/cureus.74383

**Published:** 2024-11-25

**Authors:** Khin Yadanar Kyaw, Min Thant Lwin, Zaw Thant Lwin

**Affiliations:** 1 Internal Medicine, University Hospitals of Derby and Burton NHS Foundation Trust, Derby, GBR; 2 Acute Medicine, University Hospitals of Derby and Burton NHS Foundation Trust, Derby, GBR

**Keywords:** functional vitamin b12 deficiency, laughing gas, neurology case report, nitrous oxide abuse, sacd

## Abstract

Nitrous oxide (N_2_O) is generally used in the medical and food industries. However, it is sometimes illegally misused by young adults as a recreational drug. In either of these uses, functional vitamin B12 deficiency results in neurological implications, such as peripheral neuropathy and subacute combined degeneration (SACD). Here, we report a case of N_2_O-induced SACD, in which a diagnosis of functional B12 deficiency was made based on borderline normal serum vitamin B12 levels with elevated metabolites (methylmalonic acid and homocysteine). In this case, treatment with intramuscular (IM) vitamin B12 led to significant clinical improvement.

## Introduction

Nitrous oxide (N_2_O), also known as “laughing gas” or “whippets,” is commonly used in the medical field as an anaesthetic and in the food industry as a foaming agent [1,2]. However, it is increasingly misused by young adults as a recreational drug, owing to its easy accessibility and its effects, which include euphoria, relaxation and calmness [1,2]. On the downside, N_2_O can cause adverse effects such as hallucinations, arrhythmias, seizures, falls and vitamin B12 deficiency, leading to peripheral neuropathy and subacute combined degeneration (SACD) of the spinal cord [1-3]. According to data from the Office for National Statistics (ONS), the UK government reported that recreational usage of N_2_O among the 16-59 age group in England and Wales was 1.3% in 2021-2022. In Scotland, 3% of the 16-24 age group, 1% of the 25-35 age group and 1% of the 35-44 age group were reported to use N_2_O as a recreational drug in 2020. Between 2018 and 2020, N_2_O was the third most-used recreational drug in England and Wales [4]. In November 2023, the UK government announced that it is a criminal offence to possess and use N_2_O to get its euphoric effect [5].

“This article was previously presented as a poster at the Royal College of Physicians and Surgeons, Glasgow - Medicine 2024 conference on October 24-25, 2024.”

## Case presentation

A 21-year-old female, who was fit and well with no underlying medical conditions, presented to our medical same-day emergency care (Medical SDEC) with a two-month history of progressive numbness and weakness. It started on the tips of her fingers and toes and later progressed to her mid-calves and mid-thighs. She also mentioned that walking had been more difficult; she was unable to push down the car pedals and had to pull over to the side of the road during that period. She did not report experiencing visual problems, fever, rash, incontinence or respiratory muscle weakness. Additionally, she reported using 16 canisters of N_2_O for recreation twice a month for six months and, two weeks prior, she reported using 12 canisters (each canister is about 660 grams).

On neurological examination, the sensation was reduced in a glove-and-stocking pattern, extending up to L3 and L5 in both legs. Muscle power was reduced on right knee flexion and extension, otherwise normal in both upper and lower limbs. No abnormality was found on cranial nerve examination.

**Figure 1:**
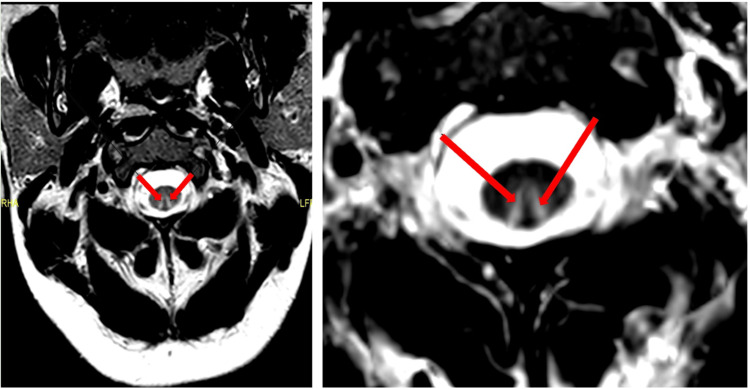
Axial T2W turbo spin echo sequences of the cervical spine Images show a symmetric, high, upside-down V-shaped signal in the dorsal columns on either side of the midline from C2 to C6 levels (sagittal imaging does not show this due to partial volume artefact, hence it is only seen reliably on the axials)

Her initial blood tests showed normal full blood count, urea, electrolytes, liver function tests, borderline vitamin B12 (208 ng/L; reference range: 197-771 ng/L) and low folate (3.3 μg/L; reference range: 3.8-26.8 μg/L) (Table 1). An urgent MRI of the whole spine was performed, revealing a symmetric, high, upside-down V-shaped signal in the dorsal columns on either side of the midline from C2 to C6 levels (Figure 1). The findings were consistent with SACD of the spinal cord. After consulting with the neurology team, methylmalonic acid (MMA) and homocysteine levels were also included in the blood tests. Her nerve conduction study (NCS) showed evidence of a large-fibre sensory motor axonal polyneuropathy with subacute denervation distally evident on electromyography (EMG) in the right leg. In addition, there was a mild slowing of motor conduction velocities in the lower limbs, which supported the diagnosis of SACD. Her NCS/EMG was provided with only the report, without figures, from our neurophysiology department.

**Table 1:** Blood results g/L, Grams per litre; MCV, Mean corpuscular volume; fL, Femtoliters; ng/L, Nanograms per litre; μg/L, Micrograms per litre; nmol/L, Nanomoles per litre; μmol/L, Micromoles per litre

Test	Result
Haemoglobin	123 g/L (120-150 g/L)
Mean corpuscular volume (MCV)	88.4 fL (83-100 fL)
Vitamin B12	208 ng/L (197-771 ng/L)
Folate	3.3 μg/L (3.8-26.8 μg/L)
Methylmalonic acid (MMA)	6050 nmol/L (0-280 nmol/L)
Homocysteine	63 μmol/L (0-15 μmol/L)

She was admitted to the medical ward for observation of her neurological symptoms and physiotherapy assessment. She stayed for 17 days, then she was discharged safely. During her admission, she was given intramuscular (IM) hydroxocobalamin 1 mg every alternate day for two weeks, followed by every three months, along with folic acid supplements.

A few weeks after her discharge, her MMA level returned to 6050 nmol/L (0-280 nmol/L), and homocysteine was 63 μmol/L (0-15 μmol/L) (Table 1). In this case, it was confirmed that she had functional B12 deficiency due to N_2_O, resulting in peripheral neuropathy and SACD. At her neurology follow-up, five to six months later, she showed significant neurological improvement. She was further educated on the importance of avoiding N_2_O, as well as advised on dietary sources of vitamin B12 and folate. Plans were made to refer her for further rehabilitation if she continued to experience difficulties with daily tasks.

## Discussion

**Figure 2:**
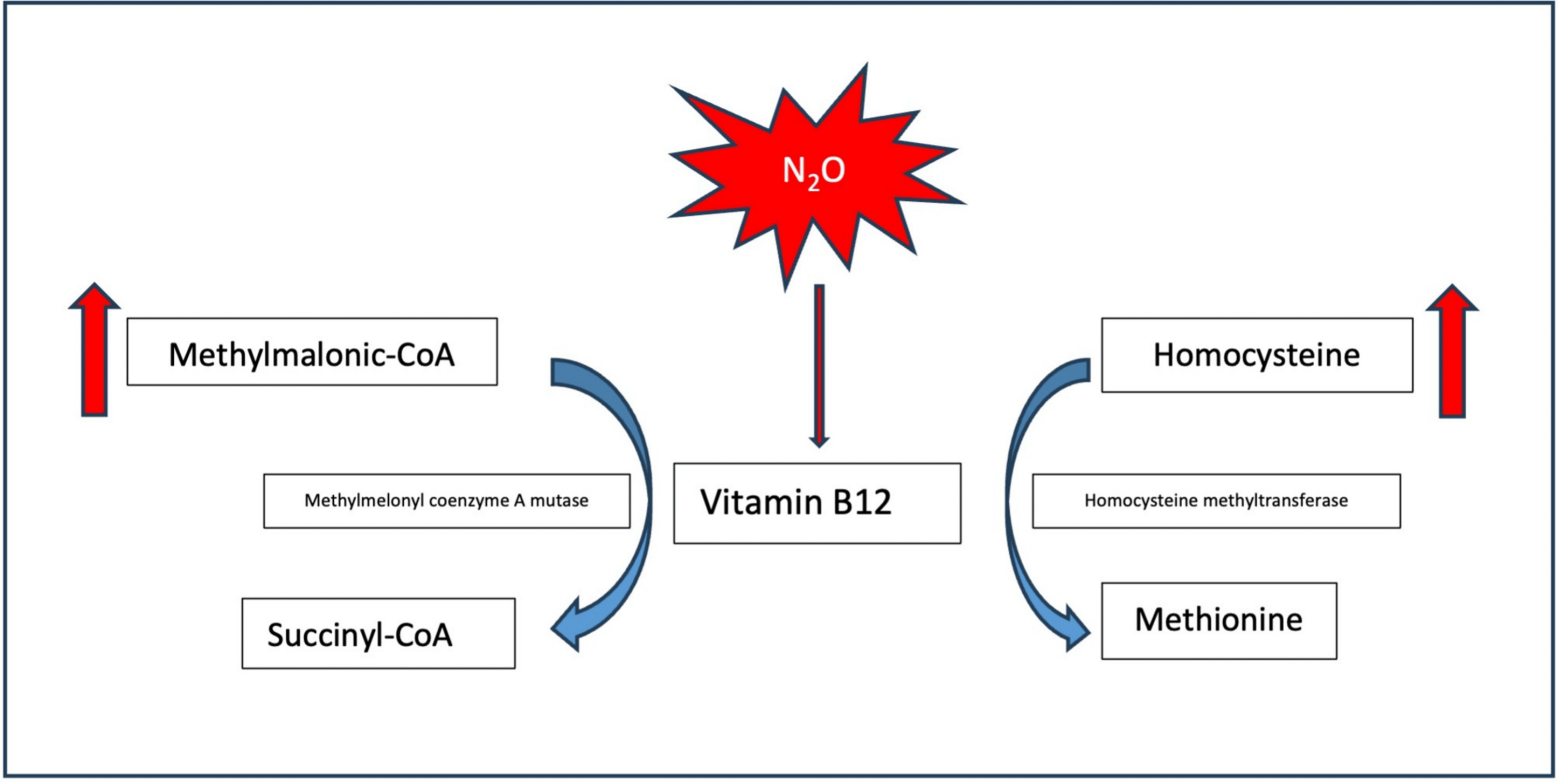
Alterations in biochemical pathways of vitamin B12 induced by nitrous oxide (N2O) Vitamin B12 serves as a cofactor of homocysteine methyltransferase, which converts homocysteine to methionine, and as a co-factor for methylmalonyl-coenzyme A mutase, which converts methylmalonyl-coenzyme A to succinyl coenzyme A (Succinyl-CoA) [7]

The main source of human vitamin B12 is a dietary source, which includes a group of compounds known as cobalamins. Along with folic acid, cobalamins serve as vital co-factors for DNA synthesis, which is essential for neuronal myelination [6]. Vitamin B12 serves as a cofactor of homocysteine methyltransferase, which converts homocysteine to methionine and is important for sustaining the integrity of the nerve sheath. It also acts as a co-factor for methylmalonyl-coenzyme A mutase, which converts methylmalonyl-coenzyme A to succinyl coenzyme A, a key step in myelin synthesis (Figure 2) [7]. 

There are many causes of vitamin B12 deficiency along its metabolic pathway, including dietary intake, malabsorption, gastrointestinal (GI) disease, pancreatic insufficiency, pernicious anaemia and N_2_O toxicity. Vitamin B12 deficiency states can be defined as the absolute depletion of vitamin B12 levels in the serum or certain pathological conditions that block its functionality to serve as a cofactor, known as functional vitamin B12 deficiency. This deficiency state can lead to systemic, haematological and neurological implications [8]. Neurological symptoms can appear before the haematological abnormalities [7].

In this discussion, we will be focusing on N_2_O toxicity. N_2_O disrupts the function of vitamin B12 by inactivating it through oxidation, causing it to become ineffective and dysfunctional as a cofactor in metabolic pathways (Figure 2) [1,9]. This results in functional vitamin B12 deficiency and, consequently, neurological symptoms, such as peripheral neuropathy, visual and gait disturbances, SACD, depression and dementia [1,7,9].

Interestingly, a study shows that one-third of patients with SACD have normal or high levels of serum B12 [7]. As serum vitamin B12 levels are calculated by immunoassays using intrinsic factors, it is not able to differentiate between functional B12 and its oxidized form [9]. Therefore, in these patients with normal B12 levels, serum metabolites such as MMA and homocysteine levels are used to confirm functional vitamin B12 deficiency [7]. Radiologically, SACD can also be identified on an MRI of the spine, which shows hyperintensity in the dorsal columns of the cervical and upper thoracic spinal cord, known as the inverted “V” or inverted rabbit ears sign [7]. Our patient had borderline normal B12 levels without anaemia or macrocytosis. Her raised MMA and homocysteine levels, as well as MRI and NCS results, supported her diagnosis of SACD related to N_2_O-induced functional B12 deficiency.

Clinical implications related to N_2_O-induced vitamin B12 deficiency can be seen in both long-term users and those with single exposure. However, serious neurological implications are seen in only a small number of heavy users, depending on the dosage and are not seen in occasional or infrequent users [1,3]. There is no screening test available for N_2_O use currently, due to its short half-life. Therefore, obtaining a thorough history, including recreational drug use, would be valuable when evaluating patients presenting with neurological signs and symptoms [1,2].

The mainstay of treatment is to replace vitamin B12. In current clinical practice in the UK, the standard treatment for SACD is IM hydroxocobalamin 1 mg per day on alternate days for two weeks, followed by 1 mg every three months [10,11]. Educating the patient to avoid the use of N_2_O is essential for the management of N_2_O-induced SACD, as the treatment would not be effective if they continue using N_2_O [10]. Monitoring biological markers and clinical symptoms by repeating vitamin B12, MMA and homocysteine levels, as well as neurological examinations, is important in assessing the response to therapy [1,7,12]. Haematological markers can improve rapidly within weeks, while neurological symptoms seem to take at least 3 to 12 months to show improvement [7,12]. 

Additionally, in the UK, the government has issued official guidance on its website, advising that N_2_O suppliers are expected to verify the intended use of the product to ensure it is not for recreational purposes. Suppliers are encouraged to pay attention to the quantity of N_2_O purchased, the time of day it is sold and any patterns of frequent purchases by certain customers. Furthermore, a training module for teachers has been published to educate students about substances that are illegal for recreational use [13]. 

## Conclusions

The increasing recreational use of N_2_O among young adults disrupts the function of vitamin B12, leading to peripheral neuropathy and SACD. Early diagnosis, supported by detailed history taking, blood tests, imaging, treatment with vitamin supplementation and total avoidance of N_2_O, can result in a favourable prognosis and complete resolution of neurological symptoms.

Our case report highlights the importance of taking a thorough history of recreational drug use in young adults presenting with signs and symptoms of peripheral neuropathy. It also emphasizes the need to consider checking metabolic assays, such as MMA and homocysteine levels in the blood, in suspected cases of SACD with normal vitamin B12 levels.
